# Supplementary material for: Structure–function analysis of oncogenic EGFR Kinase Domain Duplication reveals insights into activation and a potential approach for therapeutic targeting
Source: Nat Commun. 2021 Mar 2;12:1382. doi: 10.1038/s41467-021-21613-6 (PMC7925532; doi:10.1038/s41467-021-21613-6)
Supplement: Supplementary file 1 — Supplementary Information [file 41467_2021_21613_MOESM1_ESM.pdf]

Supplementary Information

Structure-function analysis of oncogenic EGFR Kinase Domain Duplication reveals insights into activation and a potential approach for therapeutic targeting

Zhenfang Du[1*], Benjamin P. Brown[2,3,4*], Soyeon Kim[5], Donna Ferguson[6], Dean C. Pavlick[7], Gowtham Jayakumaran[6], Ryma Benayed[6], Jean-Nicolas Gallant[1], Yun-Kai Zhang[1], Yingjun Yan[1], Monica Red-Brewer[1], Siraj M. Ali[7], Alexa B. Schrock[7], Ahmet Zehir[6], Marc Ladanyi[6], Adam W. Smith[5], Jens Meiler[3,4,8, #], Christine M. Lovly[1,9,#]

[1]Department of Medicine, Vanderbilt University Medical Center, Nashville, TN 37232, USA.
[2]Chemical and Physical Biology Program, Vanderbilt University, Nashville, TN 37235, USA
[3]Department of Chemistry, Vanderbilt University, Nashville, TN 37235, USA.
[4]Center for Structural Biology, Vanderbilt University, Nashville, TN 37235, USA.
[5]Department of Chemistry, University of Akron, Akron, OH 44325, USA.
[6]Department of Molecular Pathology, Memorial Sloan Kettering Cancer Center, New York City, NY 10065, USA.
[7]Foundation Medicine, Inc., Cambridge, MA 02141, USA
[8]Institute for Drug Discovery, Leipzig University Medical School, Leipzig, SAC 04103, Germany
[9]Vanderbilt-Ingram Cancer Center, Vanderbilt University Medical Center, Nashville, TN 37232, USA.

*These authors contributed equally.

[#]Co-corresponding authors:

Christine M. Lovly, MD, PhD
2220 Pierce Avenue
777 Preston Research Building
Nashville, TN 37232-6307 USA
Phone 615-936-3457
E-mail: christine.lovly@vumc.org

and

Jens Meiler, PhD
465 21st Ave South
BIOSCI/MRBIII, Room 5144B
Nashville, TN 37232-8725 USA
Phone: 615-936-5662
E-mail: jens.meiler@vanderbilt.edu

This PDF file includes:

Supplementary Methods
Supplementary Figures 1 to 7
Supplementary Tables 1 and 5
Supplementary References

Supplementary Methods

Sequence data are analyzed using proprietary software developed by FMI. Sequence data are mapped to the human genome (hg19) using Burrows-Wheeler Aligner (BWA) v0.5.9[1]. PCR duplicate read removal and sequence metric collection are performed using Picard 1.47 (http://picard.sourceforge.net) and SAMtools 0.1.12a[2]. Local alignment optimization is performed using Genome Analysis Toolkit (GATK) 1.0.4705[3]. Variant calling is performed only in genomic regions targeted by the test.

Base substitution detection is performed using a Bayesian methodology, which allows for the detection of novel somatic alterations at low mutant allele frequency (MAF) and increased sensitivity for alterations at hotspot sites through the incorporation of tissue-specific prior expectations[4]. Reads with low mapping (mapping quality < 25) or base calling quality (base calls with quality ≤ 2) are discarded. Final calls are made at MAF ≥ 5% (MAF ≥ 1% at hotspots).

To detect indels, *de novo* local assembly in each targeted exon is performed using the de-Bruijn approach[5]. Key steps are:

- Collecting all read-pairs for which at least one read maps to the target region.
- Decomposing each read into constituent k-mers and constructing an enumerable graph representation (de-Bruijn) of all candidate non-reference haplotypes present.
- Evaluating the support of each alternate haplotype with respect to the raw read data to generate mutational candidates. All reads are compared to each of the candidate haplotypes via ungapped alignment, and a read ‘vote’ for each read is assigned to the candidate with best match. Ties between candidates are resolved by splitting the read vote, weighted by the number of reads already supporting each haplotype. This process is iterated until a ‘winning’ haplotype is selected.
- Aligning candidates against the reference genome to report alteration calls.

Filtering of indel candidates is carried out similarly to base substitutions, with an empirically increased allele frequency threshold at repeats and adjacent sequence quality metrics as implemented in GATK: % of neighboring bases mismatches < 25%, average neighboring base quality > 25, average number of supporting read mismatches ≤ 2. Final calls are made at MAF ≥ 5% (MAF ≥ 3% at hotspots).

Copy number alterations (CNAs) are detected using a comparative genomic hybridization (CGH)-like method. First, a log-ratio profile of the sample is acquired by normalizing the sequence coverage obtained at all exons and genome-wide SNPs (~3,500) against a process-matched normal control. This profile is segmented and interpreted using allele frequencies of sequenced SNPs to estimate tumor purity and copy number at each segment. Amplifications are called at segments with ≥ 6 copies (or ≥ 7 for triploid/≥ 8 for tetraploid tumors) and homozygous deletions at 0 copies, in samples with tumor purity ≥ 20%. Amplifications in ERBB2 are called positive at segments with ≥ 5 copies for diploid tumors.

Genomic rearrangements are identified by analyzing chimeric read pairs. Chimeric read pairs are defined as read pairs for which reads map to separate chromosomes, or at a distance of over 10 megabase (Mb). Pairs are clustered by genomic coordinate of the pairs, and clusters containing at least five chimeric pairs (three for known fusions) are identified as rearrangement candidates. Filtering of candidates is performed by mapping quality (average read mapping quality in the cluster must be 30 or above) and distribution of alignment positions. Rearrangements are annotated for predicted function (e.g., creation of fusion gene).

After completion of the Analysis Pipeline, sequence data are reviewed by trained bioinformatics personnel.

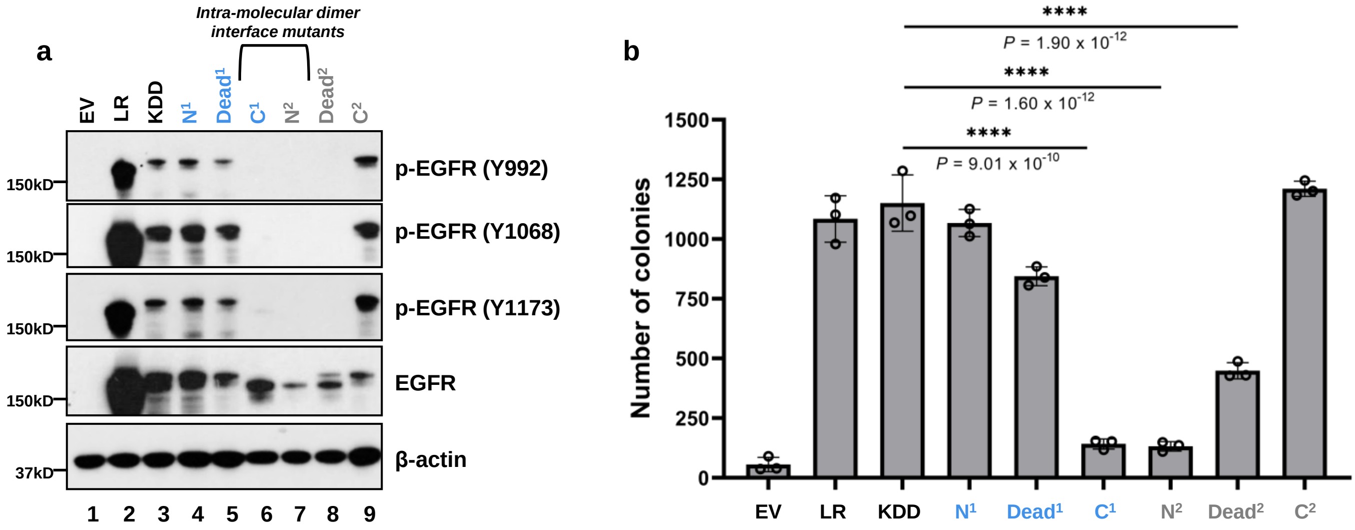

Supplementary Fig. 1 | Mutations disrupting the potential intra-molecular dimer interface abrogate the auto-phosphorylation of EGFR-KDD activation and anchorage independent growth in soft agar.

a, NR6 cells stably expressing EGFR-KDD and its mutants were cultured in serum-free medium for 48 hrs and then cells were harvested and lysed for Western blot. n=5 experiments were repeated independently with similar results (including 3 independent experiments in Supplementary Fig. 4a).

b, Anchorage-independent soft agar assays were performed in 6 well plates by seeding 5,000 NR6 in each well. n=3 biologically independent samples were examined over 3 independent experiments with similar results. Data are presented as mean values ± SD. One-Way ANOVA test with Bonferroni post hoc test was performed to obtain the adjusted P values. EV, empty vector; LR, EGFR L858R mutation.

For a and b, results are the representative of all independent experiments. Source data are provided as a Source Data file.

a

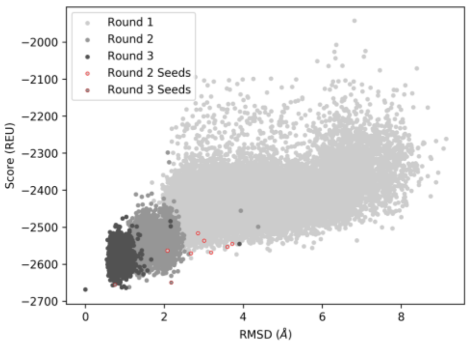

b

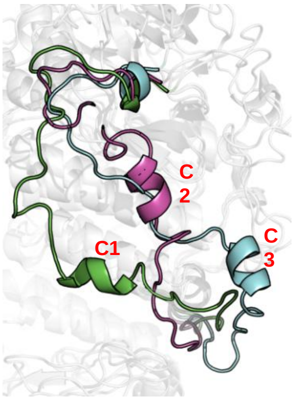

Cluster ID	Interface Score
C1	-34.86 ± 8.61
C2	-72.65 ± 8.36
C3	-47.85 ± 10.43

c

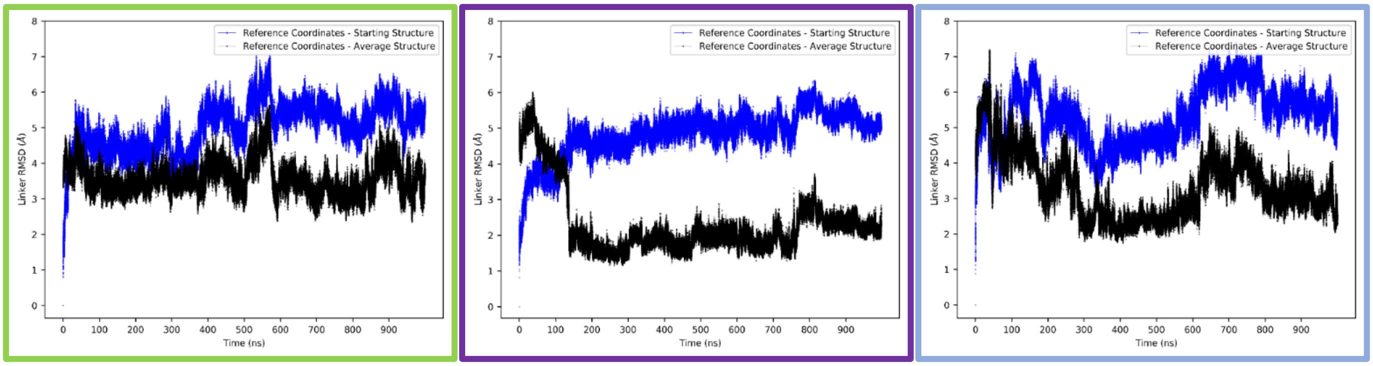

Supplementary Fig. 2 | EGFR-KDD intra-molecular dimer model building and refinement.
a, Models of the EGFR-KDD intra-molecular dimer were generated with Rosetta. Models from rounds 2 and 3 of the model building process were clustered based on the structure of the linker domain.
b, The best scoring model from each of the top three clusters (C1, green; C2, purple; C3, blue) were selected for refinement in Amber18 (left panel). Binding scores for each of the linker conformations (left panel) were computed with MM-GBSA neglecting the entropic contribution to binding (right panel). Frames for inclusion in the MM-GBSA calculation were selected every 100 ps across the entire 1.0 μs trajectory. MM-GBSA scores are represented as mean values ± SD.
c, Stability of the linker region over each 1 μs MD trajectory was analyzed by computing the RMSD of linker heavy atoms to the position of the conformation at the beginning of the production run (black trace) and the average coordinates from the whole production run (blue trace) for C1 (left panel), C2 (middle panel), and C3 (right panel).
For c, the final EGFR-KDD model is provided as Supplementary Data 2.

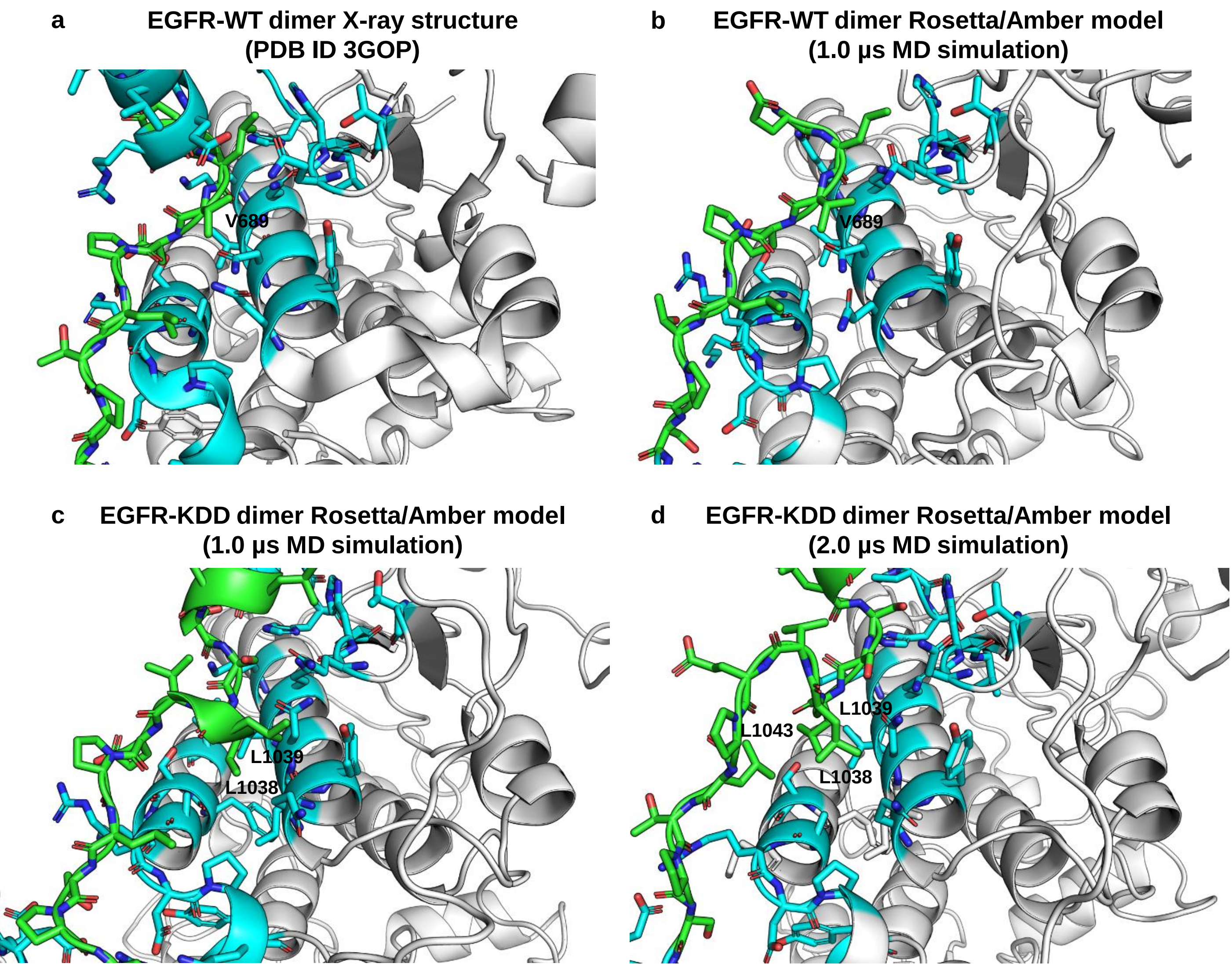

Supplementary Fig. 3 | Comparison of EGFR-KDD computational models with X-ray structure of EGFR-WT juxtamembrane latch.

a, X-ray structure of the EGFR-WT homodimer with juxtamembrane latch;

b, Rosetta model of EGFR-WT homodimer with juxtamembrane latch post-equilibration for 1.0 µs MD simulation;

c, Rosetta model of EGFR-KDD intra-molecular dimer post-equilibration for 1.0 µs MD simulation;

d, Rosetta model of EGFR-KDD intra-molecular dimer post-equilibration for 2.0 µs MD simulation; the receiver kinase domain N-terminal JMB domain is colored green; residues within 6.0 Å of JMB are colored blue.

For b, c, and d, the model coordinates are provided as Supplementary Data 1 – 3, respectively.

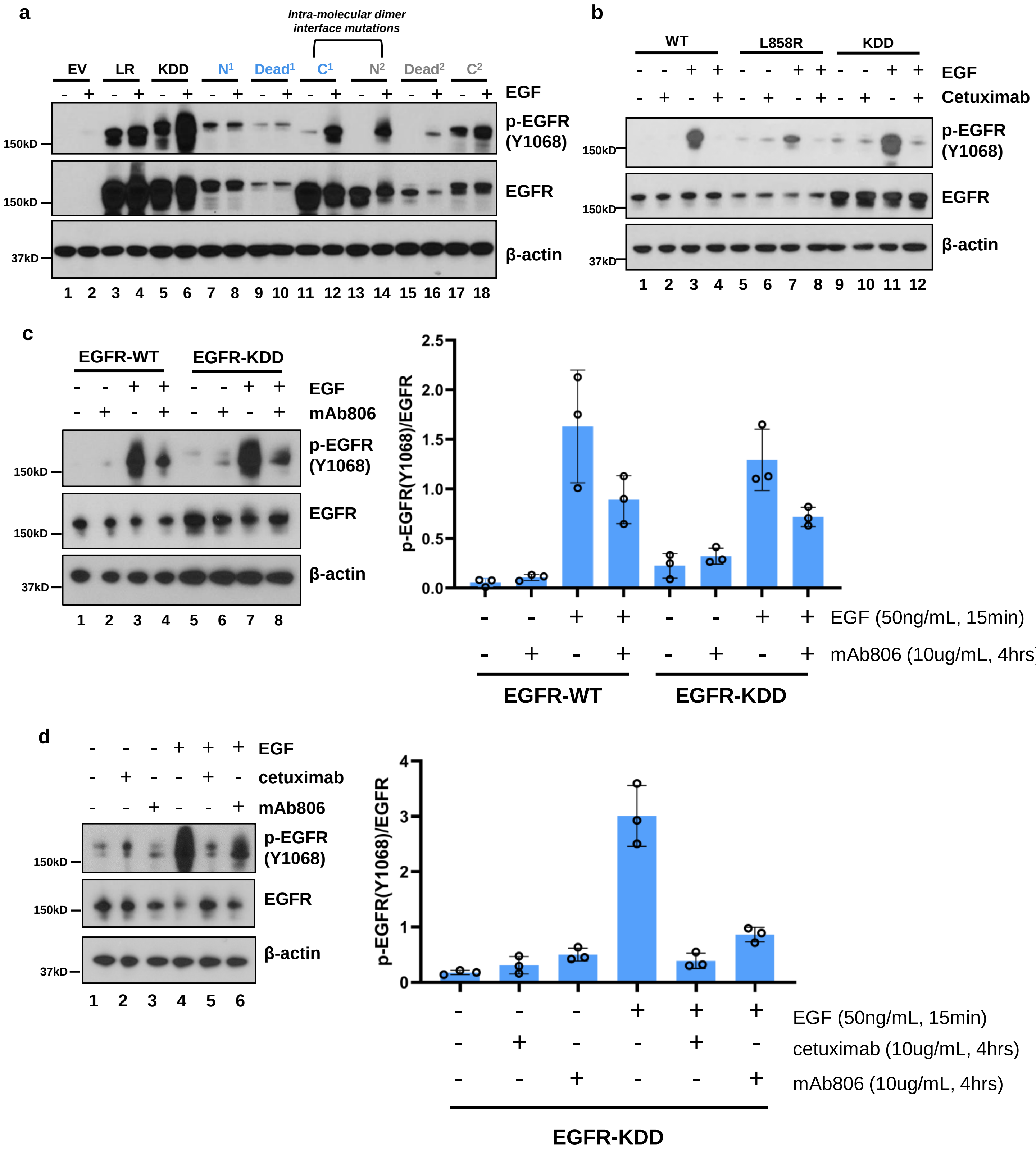
a
Intra-molecular dimer interface mutations
EV
LR
KDD
N1
Dead1
C1
N2
Dead2
C2
EGF
p-EGFR (Y1068)
EGFR
β-actin
150kD
150kD
37kD
1 2 3 4 5 6 7 8 9 10 11 12 13 14 15 16 17 18
b
WT
L858R
KDD
EGF
Cetuximab
p-EGFR (Y1068)
EGFR
β-actin
150kD
150kD
37kD
1 2 3 4 5 6 7 8 9 10 11 12
c
EGFR-WT
EGFR-KDD
EGF
mAb806
p-EGFR (Y1068)
EGFR
β-actin
150kD
150kD
37kD
1 2 3 4 5 6 7 8
p-EGFR(Y1068)/EGFR
0.0
0.5
1.0
1.5
2.0
2.5
EGF (50ng/mL, 15min)
mAb806 (10ug/mL, 4hrs)
EGFR-WT
EGFR-KDD
d
EGF
cetuximab
mAb806
p-EGFR (Y1068)
EGFR
β-actin
150kD
150kD
37kD
1 2 3 4 5 6
p-EGFR(Y1068)/EGFR
0
1
2
3
4
EGF (50ng/mL, 15min)
cetuximab (10ug/mL, 4hrs)
mAb806 (10ug/mL, 4hrs)
EGFR-KDD

Supplementary Fig. 4 | Disruption of EGF-induced inter-molecular activation of EGFR-KDD with cetuximab and mAb806.

a, NR6 cells were cultured in serum-free medium for 36 hrs and then treated with 50ng/mL EGF ligand for 5min. Total EGFR and the autophosphorylation at three tyrosine sites were assessed by Western blot.

b, NR6 cells were starved overnight and treated with cetuximab (10 µg/ml in serum-free medium) for 3hrs 45min, and then were treated with EGF (50 ng/mL in serum-free medium) and cetuximab (10 µg/ml in serum-free medium) for 15min, then cells were harvested for western blot.

c, YAMC EGFR-WT and EGFR-KDD cells were starved for 12 hrs and pre-treated with mAb806 antibody (10 µg/ml in serum-free medium) for 3hrs 45min, respectively, and EGF ligand (50 ng/mL in serum-free medium) was added for 15min. The cells were harvested and analyzed by Western blot (left panel). The ratio of phospho-EGFR (Y1068) to total EGFR expression was also shown (right panel). Results represent the mean values of three independent experiments ± SD.

d, YAMC EGFR-KDD cells were starved for 12 hrs and pre-treated with cetuximab (10 µg/ml in serum-free medium) and mAb806 antibody (10 µg/ml in serum-free medium) for 3hrs 45min, respectively, and EGF ligand (50 ng/mL in serum-free medium) was added for 15min. The cells were harvested and analyzed by Western blot (left panel). The ratio of phospho-EGFR (Y1068) to total EGFR expression was also shown (right panel). Results represent the mean values of three independent experiments ± SD.

For a - d, n=3 experiments were repeated independently with similar results. Results are the representative of three independent experiments. Source data are provided as a Source Data file.

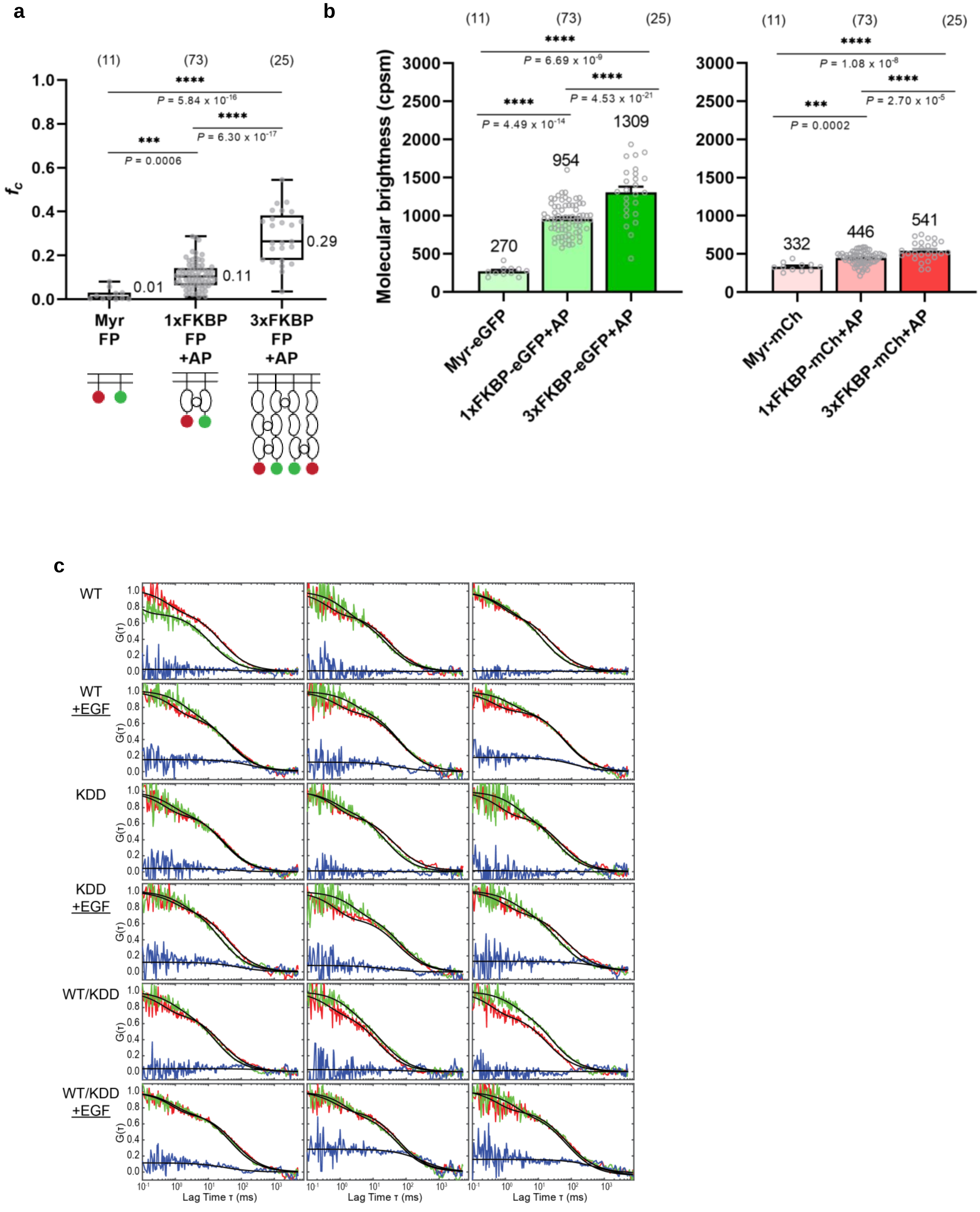
a
(11)
(73)
(25)
****
P = 5.84 x 10^-16
****
P = 6.30 x 10^-17
***
P = 0.0006
f_c
1.0
0.8
0.6
0.4
0.2
0.0
0.01
0.11
0.29
Myr FP
1xFKBP FP +AP
3xFKBP FP +AP
b
(11)
(73)
(25)
****
P = 6.69 x 10^-9
****
P = 4.53 x 10^-21
****
P = 4.49 x 10^-14
Molecular brightness (cpsm)
3000
2500
2000
1500
1000
500
0
270
954
1309
Myr-eGFP
1xFKBP-eGFP+AP
3xFKBP-eGFP+AP
(11)
(73)
(25)
****
P = 1.08 x 10^-8
****
P = 2.70 x 10^-5
***
P = 0.0002
332
446
541
Myr-mCh
1xFKBP-mCh+AP
3xFKBP-mCh+AP
c
WT
WT +EGF
KDD
KDD +EGF
WT/KDD
WT/KDD +EGF
G(τ)
Lag Time τ (ms)

Supplementary Fig. 5 | EGF ligand stimulation induces the formation of EGFR-KDD inter-molecular dimers.

a, Cross correlation values of PIE-FCCS control constructs. The monomer control (Myr-FP: myristoylated fluorescent protein [mCh or eGFP; coexpressed together]) had an fc value of 0.01 indicating no interaction. Upon cross-linking by a synthetic dimerizer (AP: AP20187) the dimer control (1xFKBP-FP) had an average f_c value of 0.11, consistent with dimerization. The multimer control (3xFKBP-FP) had an f_c value of 0.29 consistent with the formation of a mixture trimer and tetramer species.

b, Average molecular brightness of PIE-FCCS negative and positive controls in Supplementary Fig. 4c (Left: constructs with eGFP tag; right: constructs with mCh tag). The oligomer control (3xFKBP+AP) has much higher molecular brightness as expected due to clustering. mCh-tagged constructs show subtle changes in the molecular brightness due to the photophysical properties of mCherry. However, the molecular brightness changes are still statistically significant between all constructs.

c, Representative FCCS data for EGFR-WT and EGFR-KDD expressed in COS-7 cells. The scatter plot connected with red, green and blue lines indicates the normalized auto-correlation function for mCherry-fused/eGFP-fused receptors and cross-correlation function, respectively. Black solid line shows the fit model of each curves.

For a, the box and whiskers plot, the whiskers show the maximum and the minimum; the box shows 25th-75th percentile; and the line in the box is the median value. The median value is shown next to the line. For b, data are presented as mean values $\pm$ SEM. For a and b, the numbers in parentheses above the boxplot/bar graph are the total number of cells on which data were taken. One-Way ANOVA test with Uncorrected Fisher’s LSD post-hoc test was performed to obtain adjusted and individual P values. Source data are provided in the Source Data file.

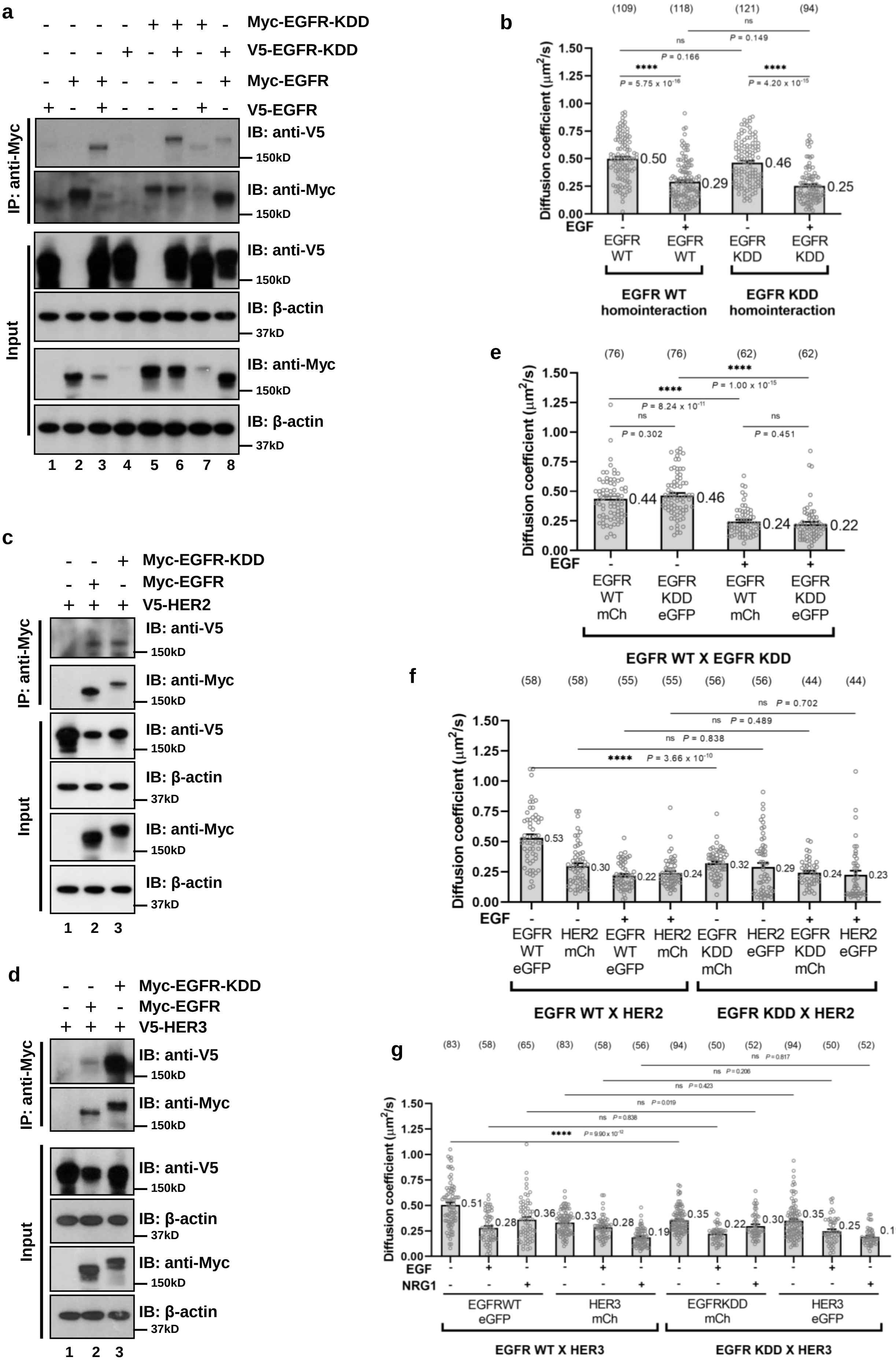
a
Myc-EGFR-KDD
V5-EGFR-KDD
Myc-EGFR
V5-EGFR
IP: anti-Myc
IB: anti-V5
150kD
IB: anti-Myc
150kD
Input
IB: anti-V5
150kD
IB: β-actin
37kD
IB: anti-Myc
150kD
IB: β-actin
37kD
1 2 3 4 5 6 7 8
b
(109) (118) (121) (94)
Diffusion coefficient (μm²/s)
ns P = 0.149
ns P = 0.166
**** P = 5.75 x 10⁻¹⁶
**** P = 4.20 x 10⁻¹⁵
0.50 0.29 0.46 0.25
EGF - + - +
EGFR WT EGFR WT EGFR KDD EGFR KDD
EGFR WT homointeraction
EGFR KDD homointeraction
c
Myc-EGFR-KDD
Myc-EGFR
V5-HER2
IP: anti-Myc
IB: anti-V5
150kD
IB: anti-Myc
150kD
Input
IB: anti-V5
150kD
IB: β-actin
37kD
IB: anti-Myc
150kD
IB: β-actin
37kD
1 2 3
d
Myc-EGFR-KDD
Myc-EGFR
V5-HER3
IP: anti-Myc
IB: anti-V5
150kD
IB: anti-Myc
150kD
Input
IB: anti-V5
150kD
IB: β-actin
37kD
IB: anti-Myc
150kD
IB: β-actin
37kD
1 2 3
e
(76) (76) (62) (62)
Diffusion coefficient (μm²/s)
**** P = 1.00 x 10⁻¹⁵
**** P = 8.24 x 10⁻¹¹
ns P = 0.302
ns P = 0.451
0.44 0.46 0.24 0.22
EGF - - + +
EGFR WT mCh
EGFR KDD eGFP
EGFR WT mCh
EGFR KDD eGFP
EGFR WT X EGFR KDD
f
(58) (58) (55) (55) (56) (56) (44) (44)
Diffusion coefficient (μm²/s)
ns P = 0.702
ns P = 0.489
ns P = 0.838
**** P = 3.66 x 10⁻¹⁰
0.53 0.30 0.22 0.24 0.32 0.29 0.24 0.23
EGF - - + + - - + +
EGFR WT eGFP
HER2 mCh
EGFR WT eGFP
HER2 mCh
EGFR KDD mCh
HER2 eGFP
EGFR KDD mCh
HER2 eGFP
EGFR WT X HER2
EGFR KDD X HER2
g
(83) (58) (65) (83) (58) (56) (94) (50) (52) (94) (50) (52)
Diffusion coefficient (μm²/s)
ns P = 0.817
ns P = 0.206
ns P = 0.423
ns P = 0.019
ns P = 0.838
**** P = 9.90 x 10⁻¹²
0.51 0.28 0.36 0.33 0.28 0.19 0.35 0.22 0.30 0.35 0.25 0.19
EGF
NRG1
EGFRWT eGFP
HER3 mCh
EGFRKDD mCh
HER3 eGFP
EGFR WT X HER3
EGFR KDD X HER3

Supplementary Fig. 6 | EGFR-KDD directly interacts with ErbB family members.

a, V5-epitope tagged EGFR-WT and EGFR-KDD was co-transfected with Myc-epitope tagged EGFR-WT and EGFR-KDD in HEK293 cells. Cell lysates were immunoprecipitated by using Myc antibody. Immunoblotting were probed by V5 and Myc antibody.

b, Average diffusion coefficient of EGFR WT homodimers with (+) or without (-) ligand (EGF) stimulation is shown (values shown in Supplementary Table 2).

c, V5-epitope tagged HER2 was co-transfected with Myc-epitope tagged EGFR-WT and EGFR-KDD in HEK293 cells. Cell lysates were immunoprecipitated by using Myc antibody. Immunoblotting were probed by V5 and Myc antibody.

d, V5-epitope tagged HER3 was co-transfected with Myc-epitope tagged EGFR-WT and EGFR-KDD in HEK293 cells. Cell lysates were immunoprecipitated by using Myc antibody. Immunoblotting were probed by V5 and Myc antibody.

e, Average diffusion coefficient of EGFR WT and EGFR KDD mutant with (+) or without (-) ligand (EGF) stimulation is shown (values shown in Supplementary Table 2).

f, Average diffusion coefficient of HER2 and EGFR-KDD mutant with (+) or without (-) ligand (EGF) stimulation is shown (values shown in Supplementary Table 2).

g, Average diffusion coefficient of HER3 and EGFR-KDD mutant with (+) or without (-) ligand (EGF or NRG1) stimulation is shown (values shown in Supplementary Table 2).

For a, c and d, n=2 experiments were repeated independently with similar results. Results are the representative of two independent experiments. Source data are provided as a Source Data file.

For b, e, f, and g, the numbers in parentheses above the bar graph are the total number of cells on which data were taken. Data are presented as mean values $\pm$ SEM. One-Way ANOVA test with Uncorrected Fisher's LSD post hoc test was performed to obtain adjusted and individual P values.

For a – g, source data are provided in the Source Data file.

a

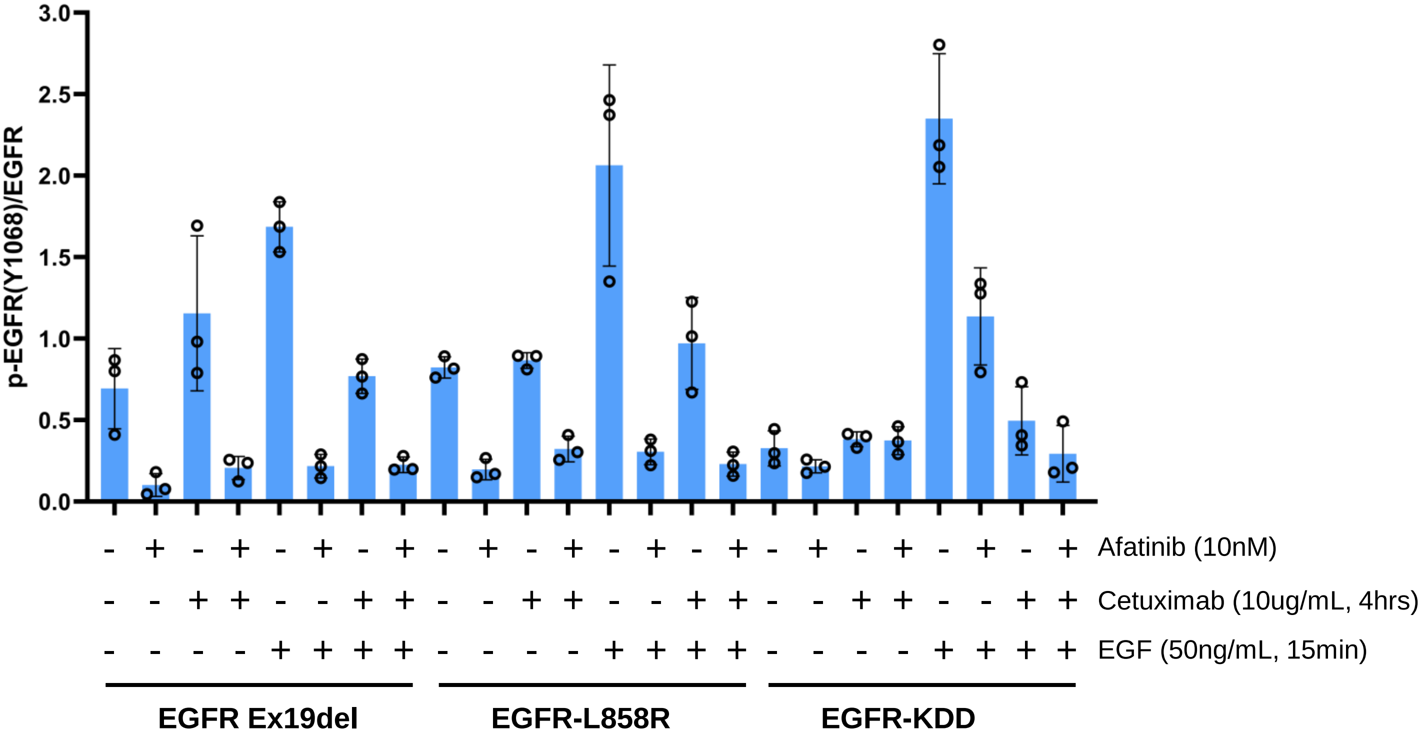

b

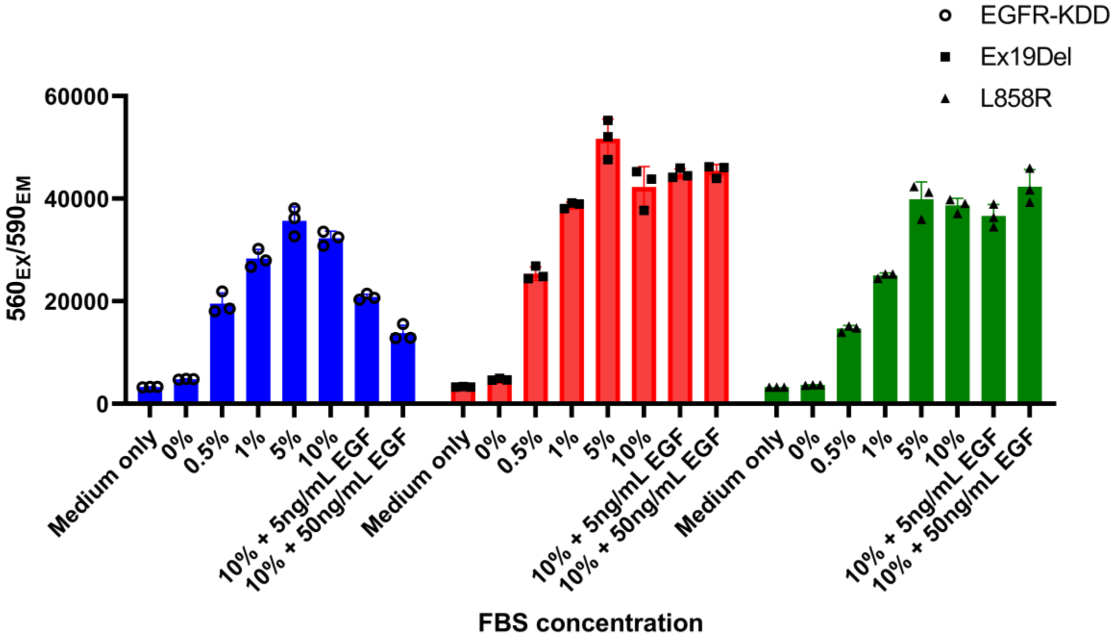

Supplementary Fig. 7

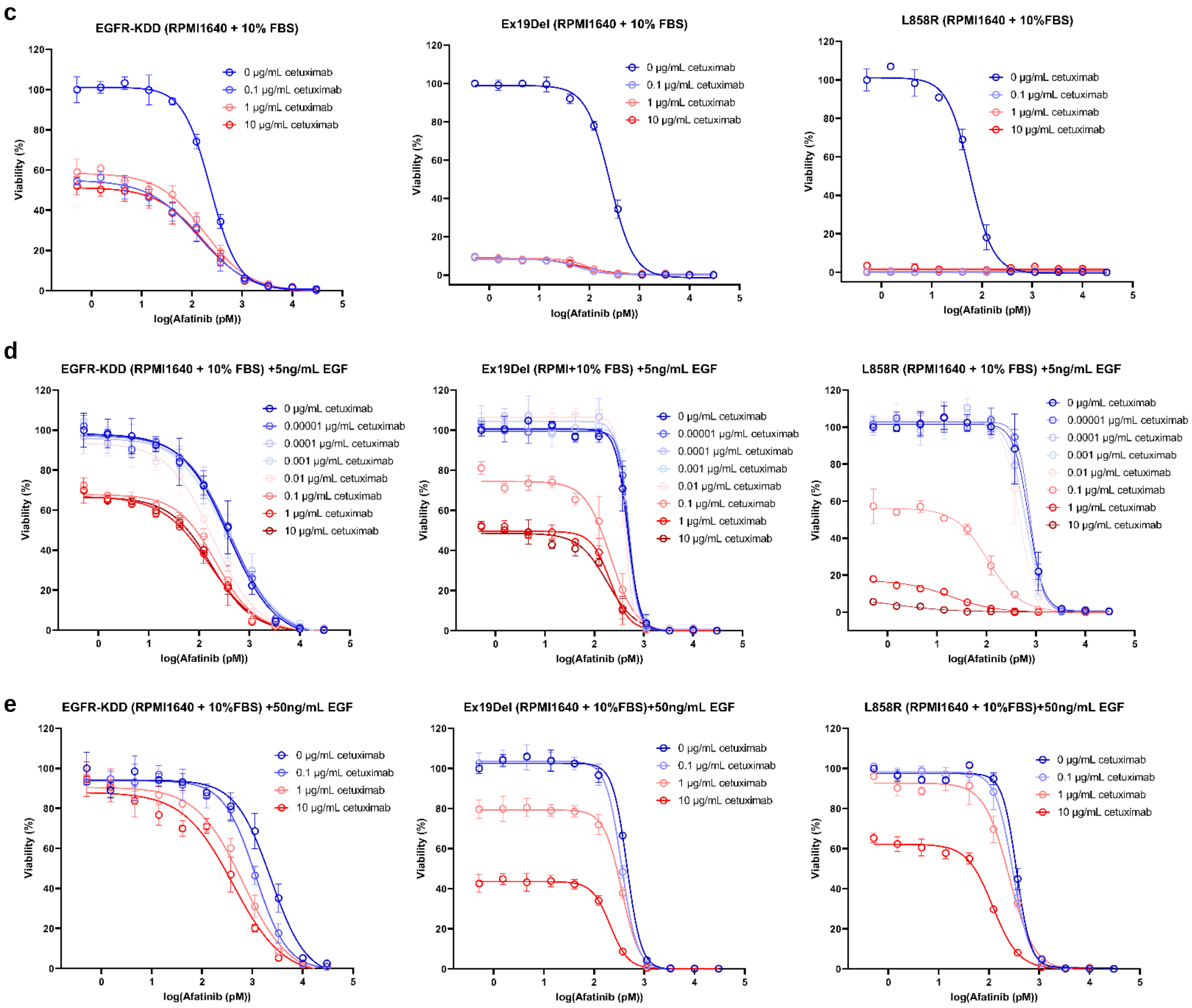

Supplementary Fig. 7 | Inhibition of EGFR-KDD is maximally achieved by blocking both intra- and inter-molecular dimerization.

a, Quantification of YAMC antibody/TKI treatment Western blots in Fig. 5a. n=3 experiments were repeated independently with similar results.

b, BaF3 cell growth at different concentration of fetal bovine serum (FBS).

c, Cell Viability Assay was performed in mIL3-independent Ba/F3 cells stably expressing EGFR-KDD, Ex19Del and L858R in RPMI1640 supplemented with 10% FBS.

d, Cell Viability Assay was performed in mIL3-independent Ba/F3 cells stably expressing EGFR-KDD, Ex19Del and L858R in RPMI1640 supplemented with 10% FBS and 5ng/mL EGF.

e, Cell Viability Assay was performed in mIL3-independent Ba/F3 cells stably expressing EGFR-KDD, Ex19Del and L858R in RPMI1640 supplemented with 10% FBS and 50ng/mL EGF.

For b - e, n=3 biologically independent replicates were examined over 3 independent experiments with similar results. For a – e, data are presented as mean values ± SD. Results are the representative of three independent experiments. Source data are provided as a Source Data file.

Supplementary Tables

Supplementary Table 1 | Protein numbering of human EGFR protein in this study

Protein	Immature protein numbering[a]	Mature protein numbering[b]
EGFR-WT/EGFR-KDD	I706Q	I682Q
EGFR-WT/EGFR-KDD	D837N	D813N
EGFR-WT	L858R	L834R
EGFR-WT/EGFR-KDD	V948R	V924R
EGFR-WT	Y1016	Y992
EGFR-WT	Y1092	Y1068
EGFR-WT	Y1197	Y1173
EGFR-KDD	I1057Q	I1033Q
EGFR-KDD	D1188N	D1164N
EGFR-KDD	V1299R	V1275R
EGFR-KDD	Y1367	Y1343
EGFR-KDD	Y1443	Y1419
EGFR-KDD	Y1548	Y1524

[a] Immature, protein numbering of the human immature EGFR sequence that includes the 24-residue signal sequence;
[b] Mature, protein numbering of the human mature EGFR sequence that does not include the 24-residue signal sequence.

Supplementary Table 2 | PIE-FCCS data analysis for EGFR-WT, EGFR-KDD homodimerization, WT × KDD heterodimerization, WT × HER2 heterodimerization, and WT × HER3 heterodimerization with or without EGF/NRG1 stimulation.

	EGFR-WT		EGFR-KDD		EGFR-WT × EGFR-KDD			
Construct					EGFR-WT mCh	EGFR-KDD eGFP	EGFR-WT mCh	EGFR-KDD eGFP
EGF	-	+	-	+	-	-	+	+
Number of cells	109	121	118	94	76		62	
f_c[a]	0.027 ± 0.004	0.191 ± 0.008	0.029 ± 0.004	0.173 ± 0.009	0.045 ± 0.007		0.232 ± 0.013	
f_c[b]	0.004	0.185	0.004	0.170	0.005		0.220	
Brightness[a] (cpsm)	275 ± 9	335 ± 9	272 ± 8	275 ± 7	395 ± 10	333 ± 16	409± 13	317 ± 12
Diffusion Coefficient[a] ($\mu m^2/s$)	0.50 ± 0.02	0.29 ± 0.02	0.46 ± 0.02	0.25 ± 0.02	0.44 ± 0.02	0.46 ± 0.02	0.24 ± 0.02	0.22 ± 0.02
Density[a] ($mol/\mu m^2$)	733 ± 55	868 ± 56	484 ± 38	681 ± 52	605 ± 41	591 ± 44	574 ± 38	694 ± 50

[a]mean value; [b]median value (reported on the figures)

	EGFR-WT × HER2				EGFR-KDD × HER2			
Construct	EGFR-WT eGFP	HER2 mCh	EGFR-WT eGFP	HER2 mCh	EGFR-KDD mCh	HER2 eGFP	EGFR-KDD mCh	HER2 eGFP
EGF	-	-	+	+	-	-	+	+
Number of cells	58		55		56		44	
f_c[a]	0.048 ± 0.008		0.113 ± 0.010		0.102 ± 0.016		0.168 ± 0.016	
f_c[b]	0.007		0.105		0.058		0.159	
Brightness[a] (cpsm)	269 ± 7	320 ± 7	312 ± 13	307 ± 5	338 ± 8	233 ± 6	384 ± 10	242 ± 7
Diffusion Coefficient[a] ($\mu m^2/s$)	0.53 ± 0.03	0.30 ± 0.02	0.22 ± 0.01	0.24 ± 0.02	0.32 ± 0.02	0.29 ± 0.03	0.24 ± 0.02	0.23 ± 0.03
Density[a] ($mol/\mu m^2$)	749 ± 77	489 ± 48	907 ± 94	838 ± 63	252 ± 24	900 ± 107	359 ± 42	891 ± 92

	EGFR-WT × HER3				EGFR-KDD × HER3							
Construct	EGFR-WT eGFP	HER3 mCh	EGFR-WT eGFP	HER3 mCh	EGFR-WT eGFP	HER3 mCh	EGFR-KDD mCh	HER3 eGFP	EGFR-KDD mCh	HER3 eGFP	EGFR-KDD mCh	HER3 eGFP
EGF	-	-	+	+	-	-	-	-	+	+	-	-
NRG1	-	-	-	-	+	+	-	-	-	-	+	+
Number of cells	84		58		65		94		50		52	
f_c[a]	0.047 ± 0.007		0.051 ± 0.009		0.129 ± 0.012		0.064 ± 0.006		0.089 ± 0.013		0.144 ± 0.010	
f_c[b]	0.014		0.017		0.130		0.057		0.078		0.140	
Brightness[a] (cpsm)	260 ± 7	383 ± 10	377 ± 13	330 ± 8	271 ± 6	385 ± 12	319 ± 5	292 ± 8	387 ± 12	287 ± 10	302 ± 7	297 ± 10
Diffusion Coefficient[a] ($\mu m^2/s$)	0.51 ± 0.02	0.33 ± 0.01	0.28 ± 0.02	0.28 ± 0.01	0.36 ± 0.03	0.19 ± 0.01	0.35 ± 0.01	0.35 ± 0.02	0.22 ± 0.01	0.25 ± 0.02	0.30 ± 0.02	0.19 ± 0.01
Density[a] ($mol/\mu m^2$)	433 ± 27	258 ± 19	438 ± 42	379 ± 36	430 ± 39	393 ± 46	169 ± 18	232 ± 19	186 ± 17	313 ± 30	212 ± 25	257 ± 26

Supplementary Table 3 | Viability of BaF3 EGFR-KDD, Ex19Del and L858R cells at different concentration of cetuximab (N=3[a], mean ± SD)

Cetuximab (μg/mL)	RPMI1640 + 0.5% FBS			RPMI1640 + 10% FBS			RPMI1640 + 10% FBS + 5ng/mL EGF			RPMI1640 + 10% FBS + 50ng/mL EGF		
	EGFR-KDD	Ex19Del	L858R	EGFR-KDD	Ex19Del	L858R	EGFR-KDD	Ex19Del	L858R	EGFR-KDD	Ex19Del	L858R
0	100	100	100	100	100	100	100	100	100	100	100	100
0.00001	102.23 ± 1.65	104.87 ± 6.02	100.19 ± 4.45	97.54 ± 1.30	101.72 ± 9.61	96.86 ± 7.91	100.20 ± 2.08	102.09 ± 1.04	103.57 ± 4.26	NT	NT	NT
0.0001	104.60 ± 5.34	102.62 ± 7.75	96.96 ± 4.29	92.74 ± 5.78	95.38 ± 3.52	101.25 ± 3.11	95.21 ± 2.99	99.41 ± 4.60	102.04 ± 4.98	NT	NT	NT
0.001	110.95 ± 7.60	100.63 ± 10.90	91.00 ± 7.40	92.66 ± 15.99	95.90 ± 9.68	101.46 ± 5.11	95.75 ± 9.24	99.52 ± 3.51	104.71 ± 1.50	NT	NT	NT
0.01	89.24 ± 4.75	42.65 ± 13.74	15.15 ± 13.26	66.71 ± 11.73	68.01 ± 14.09	50.62 ± 18.57	88.87 ± 6.71	100.71 ± 2.31	100.54 ± 7.81	NT	NT	NT
0.1	60.95 ± 5.35	39.66 ± 18.78	1.75 ± 0.37	49.12 ± 9.82[a]	19.72 ± 10.04[a]	0.48 ± 0.32[a]	70.98 ± 3.78	84.88 ± 3.29	69.87 ± 20.42	104.06 ± 13.59	101.55 ± 0.99	102.33 ± 2.41
1.0	57.05 ± 1.66	34.14 ± 14.27	2.83 ± 2.33	45.29 ± 7.25[a]	12.34 ± 3.44	0.46 ± 0.25	67.53 ± 2.15	44.96 ± 6.80	17.24 ± 5.30	104.62 ± 7.79	78.66 ± 3.06	90.93 ± 8.60
10	61.01 ± 2.54	35.19 ± 14.37	2.83 ± 2.19	45.84 ± 6.49[a]	12.67 ± 5.07	1.45 ± 1.85	68.66 ± 1.23	46.31 ± 6.60	5.11 ± 2.60	104.81 ± 9.74	46.59 ± 4.58	66.04 ± 4.68

NT, not tested; a, N=6

Supplementary Table 4 | EC50 of afatinib (nM) in different concentration of cetuximab in BaF3 cells (N=3[a], mean ± SD)

Cetuximab (μg/mL)	RPMI1640 + 0.5% FBS			RPMI1640 + 10% FBS			RPMI1640 + 10% FBS + 5ng/mL EGF			RPMI1640 + 10% FBS + 50ng/mL EGF		
	EGFR-KDD	Ex19Del	L858R	EGFR-KDD	Ex19Del	L858R	EGFR-KDD	Ex19Del	L858R	EGFR-KDD	Ex19Del	L858R
0	0.103 ± 0.035	0.060 ± 0.017	0.020 ± 0.013	0.456 ± 0.168[a]	0.340 ± 0.236[a]	0.088 ± 0.043[a]	0.357 ± 0.119	0.410 ± 0.062	0.466 ± 0.242	1.762 ± 1.715	0.951 ± 1.033	0.626 ± 0.551
0.00001	0.101 ± 0.009	0.069 ± 0.033	0.024 ± 0.019	0.344 ± 0.132	0.243 ± 0.201	0.103 ± 0.049	0.358 ± 0.107	0.408 ± 0.082	0.525 ± 0.212	NT	NT	NT
0.0001	0.104 ± 0.021	0.061 ± 0.008	0.020 ± 0.015	0.463 ± 0.135	0.240 ± 0.178	0.101 ± 0.039	0.349 ± 0.114	0.401 ± 0.046	0.448 ± 0.164	NT	NT	NT
0.001	0.077 ± 0.021	0.060 ± 0.019	0.021 ± 0.019	0.269 ± 0.135	0.229 ± 0.175	0.085 ± 0.038	0.333 ± 0.067	0.420 ± 0.076	0.483 ± 0.115	NT	NT	NT
0.01	0.027 ± 0.009	0.052 ± 0.025	0.002 ± 0.002	0.208 ± 0.063	0.128 ± 0.083	0.013 ± 0.012	0.235 ± 0.081	0.331 ± 0.075	0.353 ± 0.118	NT	NT	NT
0.1	0.104 ± 0.050	0.062 ± 0.027	0.002 ± 0.003	0.267 ± 0.110[a]	0.110 ± 0.087[a]	0.003 ± 0.002	0.220 ± 0.044	0.143 ± 0.059	0.118 ± 0.024	0.832 ± 0.803	0.453 ± 0.451	0.362 ± 0.310
1.0	0.098 ± 0.013	0.050 ± 0.017	0.001 ± 0.002	0.361 ± 0.104[a]	0.207 ± 0.157[a]	NA	0.183 ± 0.086	0.150 ± 0.055	0.025 ± 0.020	0.501 ± 0.476	0.630 ± 0.674	0.404 ± 0.366
10	0.095 ± 0.040	0.061 ± 0.027	0.003 ± 0.004	0.273 ± 0.102[a]	0.095 ± 0.045[a]	NA	0.189 ± 0.071	0.140 ± 0.039	0.011 ± 0.010	0.303 ± 0.293	0.204 ± 0.203	0.168 ± 0.134

NA, EC50 is not available due to the invalid drug dose-response curves.
NT, not tested.
a, N=6

Supplementary Table 5 | Primers used for plasmids construction in the present study

Primers	Sequence (5'-3')
KDD-Tag-F	agtgatgtctggagctacggggtgacc
KDD-Tag-R	ggaccggtCTCGAGCGGCCGCCtgctccaataaattcactgctttgtggcgcgacc
	CCGGTCGCCACCGGTAAGCCTATCCCTAACCCTCTCCTCGGTCTCGATTCTACGCG
V5HisB-for EGFP-F	TACCGGTCATCATCACCATCACCATTGAAGC
	GGCCGCTTCAATGGTGATGGTGATGATGACCGGTACGCGTAGAATCGAGACCGAG
V5HisB-for EGFP-R	GAGAGGGTTAGGGATAGGCTTACCGGTGGCGA
	CCGGTCGCCACCGAACAAAAACTCATCTCAGAAGAGGATCTGAATATGCATACCGG
MycHisB-for EGFP-F	TCATCATCACCATCACCATTGAAGC
	GGCCGCTTCAATGGTGATGGTGATGATGACCGGTATGCATATTCAGATCCTCTTCT
MycHisB-for EGFP-R	GAGATGAGTTTTTGTTCGGTGGCGA
KDD-L1038A-F	GACTCCCCTCCTGAGCTCTGCGCTTGTGGAGCCTCTTACACCCAG
KDD-L1038A-R	CTGGGTGTAAGAGGCTCCACAAGCGCAGAGCTCAGGAGGGGAGTC
KDD-L1039A-F	GACTCCCCTCCTGAGCTCTCTGGCTGTGGAGCCTCTTACACCCAG
KDD-L1039A-R	CTGGGTGTAAGAGGCTCCACAGCCAGAGAGCTCAGGAGGGGAGTC
KDD-LLAA-F	GACTCCCCTCCTGAGCTCTGCGGCTGTGGAGCCTCTTACACCCAG
KDD-LLAA-R	CTGGGTGTAAGAGGCTCCACAGCCGCAGAGCTCAGGAGGGGAGTC
KDD-L1038R-FN	GACTCCCCTCCTGAGCTCTCGCCTTGTGGAGCCTCTTACACCCAG
KDD-L1038R-RN	CTGGGTGTAAGAGGCTCCACAAGGCGAGAGCTCAGGAGGGGAGTC
KDD-L1039R-FN	GACTCCCCTCCTGAGCTCTCTGCGCGTGGAGCCTCTTACACCCAG
KDD-L1039R-RN	CTGGGTGTAAGAGGCTCCACGCGCAGAGAGCTCAGGAGGGGAGTC
KDD-LLRR-FN	GACTCCCCTCCTGAGCTCTCGCCGCGTGGAGCCTCTTACACCCAG
KDD-LLRR-RN	CTGGGTGTAAGAGGCTCCACGCGGCGAGAGCTCAGGAGGGGAGTC
KDD-(GGS)$_3$-F	CACGGACTCCCCTCCTGAGCTCTGGAGGAAGTGGAGGAAGTGGAGGAAGTGGAGA
	AGCTCCCAACCAAGCTC
KDD-(GGS)$_3$-R	GAGCTTGGTTGGGAGCTTCTCCACTTCCTCCACTTCCTCCACTTCCTCCAGAGCTC
	AGGAGGGGAGTCCGTG
KDD-(GGS)$_6$-F	CGACGAGTACCTCATCCCAGGAGGAAGTGGAGGAAGTGGAGGAAGTGGAGGAAGT
	GGAGGAAGTGGAGGAAGTCTGCTTGTGGAGCCTCTTACACCCAG
KDD-(GGS)$_6$-R	CTGGGTGTAAGAGGCTCCACAAGCAGACTTCCTCCACTTCCTCCACTTCCTCCACT
	TCCTCCACTTCCTCCACTTCCTCCTGGGATGAGGTACTCGTCG
Seq-2R	AGCAGTCACTGGGGGACTT
Seq-2F	GTGATCCAAGCTGTCCCAAT
Seq-4F	TGGATCCACAGGAACTGGAT
Seq-6F	GTGTGCCCACTACATTGACG
Seq-8F	AGCTCATCACG CAGCTCAT
Seq-10F	GAATGCATTTGCCAAGTCCT

Supplementary References:

1. Li, H. & Durbin, R. Fast and accurate long-read alignment with Burrows-Wheeler transform. *Bioinformatics* **26**, 589-595 (2010).
2. Li, H. et al. The Sequence Alignment/Map format and SAMtools. *Bioinformatics* **25**, 2078-2079 (2009).
3. DePristo, M.A. et al. A framework for variation discovery and genotyping using next-generation DNA sequencing data. *Nat Genet* **43**, 491-498 (2011).
4. Forbes, S.A. et al. COSMIC: mining complete cancer genomes in the Catalogue of Somatic Mutations in Cancer. *Nucleic Acids Res* **39**, D945-950 (2011).
5. Compeau, P.E., Pevzner, P.A. & Tesler, G. How to apply de Bruijn graphs to genome assembly. *Nat Biotechnol* **29**, 987-991 (2011).
