# Supplementary material for: Structure–function analysis of oncogenic EGFR Kinase Domain Duplication reveals insights into activation and a potential approach for therapeutic targeting
Source: Nat Commun. 2021 Mar 2;12:1382. doi: 10.1038/s41467-021-21613-6 (PMC7925532; doi:10.1038/s41467-021-21613-6)
Supplement: Supplementary file 3 — Description of Additional Supplementary Files [file 41467_2021_21613_MOESM3_ESM.docx]

**Descriptions of Additional Supplementary Files**

**File Name: Supplementary Data 1**

Description: PDB coordinates of the EGFR-WT inter-molecular kinase domain dimer model after 1 microsecond MD simulation.

**File Name: Supplementary Data 2**

Description: PDB coordinates of the EGFR-KDD intra-molecular kinase domain dimer model after 1 microsecond MD simulation.

**File Name: Supplementary Data 3**

Description: PDB coordinates of the EGFR-KDD intra-molecular kinase domain dimer model after 2 microseconds MD simulation.
