# Supplementary material for: Structure–function analysis of oncogenic EGFR Kinase Domain Duplication reveals insights into activation and a potential approach for therapeutic targeting
Source: Nat Commun. 2021 Mar 2;12:1382. doi: 10.1038/s41467-021-21613-6 (PMC7925532; doi:10.1038/s41467-021-21613-6)
Supplement: Supplementary file 7 — Reporting Summary [file 41467_2021_21613_MOESM7_ESM.pdf]

nature research

Corresponding author(s): Christine M. Lovly and Jens Meiler

Last updated by author(s): Jan 27, 2021

Reporting Summary

Nature Research wishes to improve the reproducibility of the work that we publish. This form provides structure for consistency and transparency in reporting. For further information on Nature Research policies, see our Editorial Policies and the Editorial Policy Checklist.

Statistics

For all statistical analyses, confirm that the following items are present in the figure legend, table legend, main text, or Methods section.

n/a	Confirmed	
☐	☒	The exact sample size (n) for each experimental group/condition, given as a discrete number and unit of measurement
☐	☒	A statement on whether measurements were taken from distinct samples or whether the same sample was measured repeatedly
☐	☒	The statistical test(s) used AND whether they are one- or two-sided<br>*Only common tests should be described solely by name; describe more complex techniques in the Methods section.*
☒	☐	A description of all covariates tested
☒	☐	A description of any assumptions or corrections, such as tests of normality and adjustment for multiple comparisons
☐	☒	A full description of the statistical parameters including central tendency (e.g. means) or other basic estimates (e.g. regression coefficient) AND variation (e.g. standard deviation) or associated estimates of uncertainty (e.g. confidence intervals)
☐	☒	For null hypothesis testing, the test statistic (e.g. F, t, r) with confidence intervals, effect sizes, degrees of freedom and P value noted<br>*Give P values as exact values whenever suitable.*
☒	☐	For Bayesian analysis, information on the choice of priors and Markov chain Monte Carlo settings
☒	☐	For hierarchical and complex designs, identification of the appropriate level for tests and full reporting of outcomes
☒	☐	Estimates of effect sizes (e.g. Cohen's d, Pearson's r), indicating how they were calculated

Our web collection on statistics for biologists contains articles on many of the points above.

Software and code

Policy information about availability of computer code

Data collection	ImageJ Software, GelCount (Oxford Optronix), Synergy HTX microplate reader (BioTek Instruments, Winooski, VT, USA), MicroManager 1.4.23 (Micro-Manager:Vale Lab), Picoharp300 v2.3 (PicoQuant, Berlin, Germany), Rosetta version 3.11 (https://www.rosettacommons.org/software/academic), Amber18 (https://ambermd.org/).
Data analysis	Prism 9 (GraphPad Software), AmberTools18 (https://ambermd.org/AmberTools.php), CPPTRAJ (https://github.com/Amber-MD/cpptraj), MATLAB R2018b (The MathWorks, Inc.), custom MatLab scripts used for processing PIE-FCCS can be made available upon request.

For manuscripts utilizing custom algorithms or software that are central to the research but not yet described in published literature, software must be made available to editors and reviewers. We strongly encourage code deposition in a community repository (e.g. GitHub). See the Nature Research guidelines for submitting code & software for further information.

Data

Policy information about availability of data

All manuscripts must include a data availability statement. This statement should provide the following information, where applicable:

- Accession codes, unique identifiers, or web links for publicly available datasets
- A list of figures that have associated raw data
- A description of any restrictions on data availability

The authors declare that all data supporting the findings of this study are available within the article and its supplementary information files or from the corresponding author upon request. The source data underlying Figs. 1c, 1d, 2d, 2e, 2g, 3a, 3b, 3e, 4a - e, 5a - c and Supplementary Figs. 1a, 1b, 4a - d, 5a - c, 6a - g and 7a - e are provided as a Source Data file. Source data are provided with this paper. Structural models underlying Figs. 1a, 2c and 2f and Supplementary Figs. 3b - d are provided as Supplementary Data 1 - 3.

The results underlying Table 1 are based on a combination of genomic sequencing data from Foundation Medicine Inc. and MSK-IMPACT. In accordance with the Health Insurance Portability and Accountability Act, in an effort to minimize the risk of re-identification of individuals, individual-level data are not publicly available. For the Foundation Medicine dataset, raw sequencing data are proprietary and not publicly available. However, requests from accredited researchers for access to de-identified individual-level or aggregate data relevant to this manuscript, such as tumor type and mutational status, can be made available upon request by contacting Dr. Alexa B. Schrock at aschrock@foundationmedicine.com. Accredited researchers should provide contact information, affiliation/organization, and research rationale. The analysis presented here from the MSK-IMPACT dataset is a re-analysis of data originally reported by Zehir and coworkers (doi: 10.1038/nm.4333). The MSK-IMPACT dataset is publicly available through the cBioPortal for Cancer Genomics (http://cbioportal.org/msk-impact). MSK-IMPACT KDD data can be made available upon request.

Protein Data Bank (PDB) identifiers 2GS6 (https://www.rcsb.org/structure/2GS6), 2ITX (https://www.rcsb.org/structure/2ITX), 3GOP (https://www.rcsb.org/structure/3GOP), and 4RIW (https://www.rcsb.org/structure/4RIW) were accessed to assist with model building for this study. In addition, Dataset 1 from the Supplementary Information of Needham and colleagues (doi: 10.1038/ncomms13307) was accessed to assist with model building of the EGFR-KDD inter-molecular dimer.

Field-specific reporting

Please select the one below that is the best fit for your research. If you are not sure, read the appropriate sections before making your selection.

☒ Life sciences ☐ Behavioural & social sciences ☐ Ecological, evolutionary & environmental sciences

For a reference copy of the document with all sections, see nature.com/documents/nr-reporting-summary-flat.pdf

Life sciences study design

All studies must disclose on these points even when the disclosure is negative.

Sample size

We did not perform any patient or in-vivo analysis as all data was generated using cell-culture standards. No statistical method was used to predetermine sample size (n).
- For cell viability assays in Ba/F3 cells, n=3 biologically independent replicates were evaluated over 3 independent experiments. This sample size is well established in the peer-reviewed literature of similar experiment (https://doi.org/10.1038/s41467-020-19579-y).
- For soft agar assays in NR6 and YAMC cells, n=3 biologically independent replicates were examined over 3 independent experiments. This sample size is well established in the peer-reviewed literature of similar experiment (https://doi.org/10.1038/s41467-020-19704-x).
- For experiments including western blots, at least 3 independent experiments were performed in each case.
- For co-immunoprecipitation experiments, 2 independent experiments were performed. We used two different antibodies to pull-down the proteins of interest.
- For PIE-FCCS, each data point was acquired in a distinct cell within prepared plates. To build the population for statistical analyses and to ensure reproducibility, data were collected across at least three separate days. Sample size is shown in each figure.
- For the Foundation Medicine dataset, 237,701 tumor samples were analyzed in this study. For MSK-IMPACT dataset, 40,165 tumor samples were analyzed in this study.

Data exclusions

Data from single cell PIE-FCCS experiments were required to meet each of the following criteria: molecular brightness had to be at least 200 cpsm to ensure the instrument alignment; eGFP/mCherry-tagged receptor local density ratio had to be between 0.5 and 1.5 to ensure the even expression of eGFP/mCherry tagged receptor proteins. For all other experiments shown, no data were excluded from the analyses or interpretations.

Replication

For cell viability assays, soft agar assays and western blots, each experiment was replicated at least 3 independent times. For co-immunoprecipitation, each experiment was replicated 2 independent times.

Randomization

Cells were assigned randomly to the experimental and control groups.

Blinding

Not applicable for this study.

Reporting for specific materials, systems and methods

We require information from authors about some types of materials, experimental systems and methods used in many studies. Here, indicate whether each material, system or method listed is relevant to your study. If you are not sure if a list item applies to your research, read the appropriate section before selecting a response.

Materials & experimental systems

n/a	Involved in the study
☐	☒ Antibodies
☐	☒ Eukaryotic cell lines
☒	☐ Palaeontology and archaeology
☒	☐ Animals and other organisms
☐	☒ Human research participants
☒	☐ Clinical data
☒	☐ Dual use research of concern

Methods

n/a	Involved in the study
☒	☐ ChIP-seq
☒	☐ Flow cytometry
☒	☐ MRI-based neuroimaging

Antibodies

Antibodies used

- EGFR (Cell Signaling, #4267), 1:2000;
- phospho-EGFR (Y992) (Cell Signaling, #2235), 1:1000;
- phospho-EGFR (Y1068) (Cell Signaling, #2234), 1:1000;
- phospho-EGFR (Y1173) (Cell Signaling, #4407), 1:1000;
- horseradish peroxidase (HRP) - conjugated anti-mouse (Cell Signaling, #7076), 1:5000;
- HRP-conjugated anti-rabbit (Cell Signaling, #7074), 1:5000;
- V5 (MCA1360GA, AbD Serotec), 1:5000;
- Myc (Sigma-Aldrich A5963); 1:5000;
- actin (Sigma-Aldrich A2066), 1:5000.

Validation

- EGFR (Cell Signaling, #4267) was validated by the manufacturer for the species reactivities with human, mouse and monkey and application in western blot (https://www.cellsignal.com/products/primary-antibodies/egf-receptor-d38b1-xp-rabbit-mab/4267);
- phospho-EGFR (Y992) (Cell Signaling, #2235) was validated by the manufacturer for the species reactivities with human, mouse and monkey and application in western blot (https://www.cellsignal.com/products/primary-antibodies/phospho-egf-receptor-tyr992-antibody/2235);
- phospho-EGFR (Y1068) (Cell Signaling, #4407) was validated by the manufacturer for the species reactivities with human, mouse and rat and application in western blot (https://www.cellsignal.com/products/primary-antibodies/phospho-egf-receptor-tyr1173-53a5-rabbit-mab/4407);
- V5 (MCA1360GA, AbD Serotec) was validated by the manufacturer for its target species as viral and application in co-Immunoprecipitation (https://www.bio-rad-antibodies.com/monoclonal/viral-v5-tag-antibody-sv5-pk1-mca1360.html?f=purified);
- Myc (Sigma-Aldrich A5963) was validated by previous studies for its species reactivities with human (PMID: 3915782) and application in co-Immunoprecipitation (PMID: 1333843).

Eukaryotic cell lines

Policy information about cell lines

Cell line source(s)

Ba/F3 cells were purchased from DSMZ. NR6 mouse fibroblasts, an NIH 3T3 line devoid of EGFR, were firstly established by Herschman lab (PMID: 302945), and were a kind gift from Dr. William Pao (PMID: 19759520). YAMC EGFR-/- cells were established by Robert H. Whitehead and Pamela S. Robinson (PMID: 17991704), and were a kind gift from Dr. Robert H. Whitehead. HEK293 cells were purchased from ATCC. Plat-GP cells were purchased from CellBioLabs. COS-7 cells were purchased from ATCC.

Authentication

Ba/F3 and HEK293 cells were authenticated by karyotyping prior to purchase. STR analysis was completed for all cell lines.

Mycoplasma contamination

All cell lines were tested for mycoplasma by PCR at least every 6 months. All cell lines were negative for Mycoplasma contamination.

Commonly misidentified lines
(See ICLAC register)

Not applicable.

Human research participants

Policy information about studies involving human research participants

Population characteristics	The Foundation Medicine (FMI) dataset included patients with cancer whose tumor samples were submitted for comprehensive genomic profiling at Foundation Medicine during routine clinical care. The majority of cases analyzed were adults (> 18 years of age) with advanced cancer, and a significant fraction of these patients are likely deceased. This is retrospective research that involves no more than a minimal risk to the privacy of patients and involves no intervention or contact with the patients. FMI provides FMI Tests at the request of treating physicians and therefore has no direct relationship with any of these patients. Moreover, in many cases the patients may no longer be associated with the treating physician who ordered their FMI Test or may be deceased, and therefore it may be impossible to contact these patients.
Recruitment	For the Foundation Medicine dataset, at the time of this retrospective analysis, patient information already existed in the Foundation Medicine genomic database for all cases.
Ethics oversight	For the Foundation Medicine dataset, approval for this study, including a waiver of informed consent and a Health Insurance Portability and Accountability Act waiver of authorization, was obtained from the Western Institutional Review Board (protocol no. 20152817).

Note that full information on the approval of the study protocol must also be provided in the manuscript.
